# Risk Assessment and Evaluation of Analytical Method of Polycyclic Aromatic Hydrocarbons (PAHs) for Deep-Fat Fried Pork Products in Korea

**DOI:** 10.3390/foods11111618

**Published:** 2022-05-30

**Authors:** Seo Yeon Kim, Hye Won Shin, Geon Hee Kim, Yong-Yeon Kim, Min-Jae Kang, Han-Seung Shin

**Affiliations:** Department of Food Science and Biotechnology, Dongguk University-Seoul, 32, Dongguk-ro, Ilsandong-gu, Goyang-si 10326, Gyeonggi-do, Korea; rlatjdus0225@naver.com (S.Y.K.); hyew301@naver.com (H.W.S.); rjshee@naver.com (G.H.K.); kimyy613@naver.com (Y.-Y.K.); sasaseu@hanmail.net (M.-J.K.)

**Keywords:** deep-fat fried pork, polycyclic aromatic hydrocarbon, risk assessment, HPLC-FLD

## Abstract

Polycyclic aromatic hydrocarbons (PAHs) are produced during incomplete combustion of organic matter. Many of them are likely to be carcinogenic and cause mutations. In this study, the PAH4 (benzo[a]pyrene (BaP), benz[a]anthracene (BaA), chrysene (CHR), benzo[b]fluoranthene (BbF)) content in deep-fat fried pork was evaluated according to temperature and time, and a risk assessment was conducted. The high performance liquid chromatography-fluorescence detection (HPLC-FLD) method for PAH4 analysis was validated by determining linearity (R^2^), recovery, limit of detection (LOD), and limit of quantitation (LOQ). The linearity was R^2^ ≥ 0.99. The PAH4 level was dependent on the temperature, time, and nature of the edible oil. Before heat treatment, the PAH4 content of pork was 0.38 μg/kg. The PAH4 content of deep-fat fried pork ranged from 0.86 to 6.86 μg/kg according to temperature (160, 180, 200 °C) and time (3, 6, 9 min). Exposure to PAH4 via the consumption of deep-fat fried pork for different age groups among the Korean population was 0.01–0.89 μg-TEQ_BaP_/kg/day, with the margin of exposure calculated as 7.88 × 10^4^–5.22 × 10^6^. The PAH4 content and risk of exposure increased proportionally with the heat treatment temperature and time. The survey provided important information in terms of evaluating the health risks that PAH compounds can cause in people’s diets due to the heat treatment of pork.

## 1. Introduction

Polycyclic aromatic hydrocarbons (PAHs) are a large class of hydrophobic organic compounds formed through the incomplete pyrolysis or combustion of organic matter. They are bio-accumulative, carcinogenic, and mutagenic contaminants and have long duration [1,2,3,4,5]. PAHs are classified as light or heavy compound through the number of fused aromatic rings in their structure. Light PAHs contain from two to four benzene rings, such as benzo[b]fluoranthene (BbF), chrysene (CHR), and benz[a]anthracene (BaA). Benzo[a]pyrene (BaP), which contains five benzene rings, is a heavy PAH. PAHs with four to six fused benzene rings can be absorbed by humans through ingestion, inhalation, or skin contact [6].

In the non-tobacco smoking and unemployed population, food is the primary source of human exposure to PAHs, occupying more than 90% of total exposure to PAHs, in contrast for smokers, the contribution from smoking may be significant [7,8]. High temperature cooked red meat, such as pork, is a source of PAHs. An increased risk of colon and rectal cancer due to the consumption of high-temperature cooked red meat is significantly reported in the literature [9,10]. Some cancers that have been associated with occupational exposure to PAHs are those of the skin and lung [11]. PAHs are ubiquitous environmental pollutants [12]. Therefore, human exposition to PAHs is practically inescapable, which proposes an important public health concern because of their recognized carcinogenic activity.

PAHs are everywhere in foodstuffs, not only as a consequence of environmental pollution, but also as a result of some thermal treatments used in the processing process of food, including frying [13,14]. Deep-fat frying involves immersing food in oil with high temperatures of 150 to 200 °C [15]. This process generates different kinds of toxicants or genotoxic material. The PAHs are formed mainly from the process of oxidizing unsaturated fatty acids at high temperatures through two processes, pyrolysis and pyrosynthesis [16].

Traditionally, BaP, the most studied and best-known PAH, was widely used as a general marker of PAHs. However, in 2008, the European Food Safety Authority (EFSA) decided that the sum of the concentration of PAH4, including BaP, BbF, CHR, and BaA, better reflects the level of PAHs in food than BaP does alone [8].

In 2011, the European Commission (EC) prolonged the scope of the regulation to include other kinds of food and to add limits for PAH4. Moreover, the latest European official food regulation regarding the maximum levels of PAHs in fats and oils intended for human consumption or used as an ingredient in food set 2 μg/kg for BaP and 10 μg/kg for PAH4 [8].

The aim of this study was to examine the influence of thermal treatment (temperature, time, types of edible oil) on the PAH4 formation in deep-fat fried pork and to perform a risk assessment by measuring the exposure to PAH4 based on the toxic equivalent (TEQ) and daily exposure assessment. High-performance liquid chromatography-fluorescence detection (HPLC-FLD) was used for precise and accurate measurements of PAH4.

## 2. Materials and Methods

### 2.1. Chemicals and Materials

The PAH4 standards, including BaA (CAS No. 56-55-3), CHR (CAS No. 218-01-9), BbF (CAS No. 205-99-2), and BaP (CAS No. 50-32-8), and 3-methylcholanthrene (CAS No. 56-49-5) used as an internal standard (IS) were purchased from Supelco (Bellefonte, PA, USA). All reagents were analytical grade, and the water was obtained from a Milli-Q water purification system (Billerica, MA, USA) (CAS No. 7732-18-5). Acetonitrile (ACN) (CAS No. 75-05-8), ethanol (CAS No. 64-17-5), n-hexane (CAS No. 110-54-3), and dichloromethane (DCM) (CAS No. 75-09-2) were purchased from Burdick & Jackson (Muskegon, MI, USA). Potassium hydroxide (KOH) (CAS No. 1310-58-3) was used for saponification. Sodium sulfate (Na_2_SO_4_) (CAS No. 7757-82-6) was used for dehydration and purchased from Junsei (Chuo-ku, Tokyo, Japan). Sep-Pak Florisil cartridges (Waters Corp., Milford, MA, USA) were used for solid-phase extraction (SPE) and purification processes. Polytetrafluoroethylene (PTFE) membrane filters (0.45 μm) were obtained from Agela Technologies (Wilmington, DE, USA).

### 2.2. Sample Preparation for Evaluation PAH4

Raw pork samples were pork neck, which purchased from different local supermarkets in the Republic of Korea. The raw meat was chopped into uniform-sized pieces (5 cm × 5 cm × 2 cm) and cooked by deep-fat frying with 500 mL of edible oils (soybean, canola, grape seed, and sunflower oil). After cooling, all of the cooked meat was homogenized in a blender, packed in plastic bags, and frozen at −20 °C until analysis.

### 2.3. Extraction and Clean-Up for Pretreatment

The homogenized processed deep-fat fried pork was allowed to stand at laboratory temperature (25 °C). Afterward, 10 g of each homogenized deep-fat fried pork product was weighed in a 300 mL round-bottomed flask and spiked with 1 mL of 100 μg/kg of 3-methylcholanthrene. A 100 mL aliquot of 1 M KOH solution in ethanol was added for alkaline saponification under reflux extraction at 80 °C for 3 h for complete hydrolysis. This process allowed for the separation of the PAHs bound to the hydrolyzed deep-fat fried pork products and eliminated the matrix that could otherwise potentially interfere with the PAHs analysis. After cooling the flask with cold water rapidly, 50 mL of ethanol−n-hexane (1:1) was added and transferred to a separating funnel (300 mL) for liquid−liquid extraction. In total, 50 mL volumes of n-hexane were used two times as the extraction solvent, followed by three times of washing with 50 mL of Milli-Q water. The eluate was filtered through filter paper (110 mm; Advantec, Toyo Roshi Kaisha, Ltd., Tokyo, Japan) and dried with 15 g of anhydrous sodium sulfate. The filtered solution was evaporated under reduced pressure at 35 °C on a rotary evaporator to 2 mL (Rotary Vacuum Evaporator N-N Series with a digital water bath, SB-100; EYELA, Tokyo, Japan). For the clean-up procedure, the SPE cartridges were conditioned by eluting with 10 mL of DCM and 20 mL of n-hexane. After the sample was loaded onto an activated SPE cartridge, it was eluted with 5 mL n-hexane, followed by 15 mL of n-hexane−DCM (3:1). The eluate was evaporated to dryness in a heating block at 37 °C under nitrogen gas. The resulting residue was re-dissolved in 1 mL ACN (Burdick & Jackson) by vortex (Scientific Industries, Inc., Bohemia, NY, USA) mixing for 1 min. The solution was refined through a 0.45-μm PTFE membrane filter and transferred to 2 mL amber screw-cap vials (Agilent Technologies, Santa Clara, CA, USA) for HPLC-FLD analysis.

### 2.4. HPLC-FLD Analysis of PAH4

The extracts were analyzed using a Dionex U3000 HPLC-FLD system (Sunnyvale, CA, USA) equipped with a Supelcosil LC-PAH column (25 cm × 4.6 mm, i.d. particle size 5 μm; Supelco) at 37 °C. The mobile phase was a mixture of ACN and Milli-Q water at 65:35 (%) in 0–20 min and 70:30 (%) in 20–60 min, and the flow rate was 1.0 mL/min. A 10 μL aliquot of the extract was automatically injected under gradient conditions. The fluorescence detector operated an excitation wavelength/emission wavelength program of 245/390 nm for 0−20 min and 294/404 nm for 20−60 min. All five concentrations of standard mixtures (PAH4 and IS) were injected at a volume of 10 μL into the HPLC-FLD to determine the calibration curve. The HPLC-FLD analysis conditions for the PAH4 are shown in Table 1. The PAH4 standards also contained 100 μg/kg of 3-methylcholanthrene as an IS for validation of the recovery values. The linear equation, coefficients of determination (R^2^), and limit of detection (LOD) and limit of quantification (LOQ) were determined. LOD and LOQ were calculated based on the standard deviation (*σ*) of the response and the slope of the calibration curve (*s*), as follows:

LOD = [3.3 × *σ*]/*s* (1)

LOQ = [10 × *σ*]/*s* (2)

### 2.5. Identification and Quantification of PAH4

PAH4 were identified by comparing their retention times with respective standards, and the IS was analyzed under the same conditions. Five concentrations (1, 2, 5, 10, and 20 μg/kg) of PAH4 solutions containing 100 µg/kg of the IS mixture were evaluated.

### 2.6. Validation of Analytical Method

The analytical method for PAH4 determination in pork was validated by determining the linearity (R^2^), LOD, LOQ, and recovery. For method validation, blank samples of pork were spiked with PAH4 (BaP, BaA, BbF, CHR) standards at different concentrations (1, 2, 5, 10, 20 µg/kg) and the IS (3-methylcholanthrene) at 100 µg/kg.

### 2.7. Application of TEQ Concentration

Exposure to PAH4 through pork consumption was determined for various age groups among the Korean population: 1–2, 3–5, 6–11, 12–18, 19–29, 30–49, 50–64, and ≥65 years.

The toxic equivalency factor (TEF) for PAHs was estimated relative to the toxicity of BaP, considering that each PAH has different toxicities. The TEQ concentration was calculated by multiplying the concentration of each PAH in pork (*Ci*) by the value of the BaP toxic equivalency factor (TEFi) for each PAH, as follows:

(3) $${\mathrm{TEQ}}_{\mathrm{BaP}}=\sum_{i=1}^{n}\left[Ci\right]\times \mathrm{TEFi}$$

### 2.8. Exposure Assessment

Dietary intake of PAHs constitutes a major source of human exposure. Human exposure is calculated through the amount of food intake, exposure period, and life expectancy in each age group, as follows:

(4) $$\mathrm{CDI}\;(\mu g/\mathrm{kg}/\mathrm{day})=\sum_{i=1}^{n}\frac{Ci\times IRi\times ED}{BW\times AT}$$

where *Ci* is the overall toxic equivalency value (TEQ) of the PAH4 in the food (µg/kg); *IRi* is the average daily intake of food according to the Korean Health Industry Development Institute (g/day); *ED* is the exposure period; *BW* is the average body weight by age group; *AT* is the average life expectancy according to Korea Statistical Information Service (KOSIS).

### 2.9. Risk Characterization

The margin of exposure (MOE) is used to assess the risk of substances not showing thresholds in a dose−response curve due to their genotoxicity and carcinogenic properties. The MOE could be one of the suitable methods for setting a priority list by comparing appropriate reference points with human intake. For carcinogenic substances, such as PAHs, an MOE above 10,000 is generally interpreted as “low concern” to public health. The MOE is calculated by dividing a benchmark dose lower confidence limit (BMDL) by the estimated chronic daily intake (CDI, µg/kg BW/day), as shown in Equation (4).

(5) $$\mathrm{MOE}=\frac{\mathrm{BMDL}}{\mathrm{CDI}}$$

In Equation (4), the BMDL value was specified by the dose–response analysis for tumor type. The BMDL for BaP and PAH4 was 70 and 340 μg/kg BW/day, respectively.

### 2.10. Statistical Analysis

All analytes were determined in triplicate, and the data were represented as mean ± standard deviation (SD). Analysis of variance (ANOVA) was conducted on data by Scheffé’s test using SAS Studio 3.8 (SAS Institute Inc., Cary, NC, USA). Significant differences were determined by *p*-values which were less than 0.05.

## 3. Results and Discussion

### 3.1. Validation and Analytical Quality Assurance for PAHs Analysis

Calibration curves were constructed by plotting the peak areas of each analyte in the standard mixture against the respective concentrations over the range of 1–20 μg/kg, as analyzed by HPLC-FID. The curves were found to be linear in the above range for each PAH4, with R^2^ ≥ 0.99.

The LOD and LOQ were established by calculating the concentration of PAH that provided a peak area with a signal-to-noise ratio (S/N) of 3 and 10, respectively. Each value was expressed as the mean of 10 measurements. In this work, the LOD ranged from 0.10 to 0.18 μg/kg, and the LOQ ranged from 0.32 to 0.55 μg/kg (Table 2). Recovery of the PAHs, measured using the peak area of the IS (3-methylcholanthrene), ranged from 88.0% to 99.9%, and the relative standard deviation (RSD) ranged from 0.2% to 1.4%.

**Figure 1:**
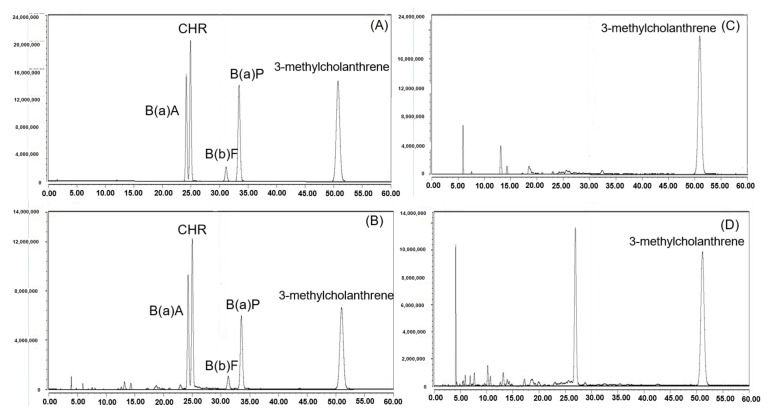
HPLC-FLD chromatograms of four PAHs standards (**A**); four PAHs with spiked sample (**B**); internal standards with blank sample (**C**); and chromatograms of PAHs for sample (**D**).

The LOD and LOQ values were lower than the performance reference values set by European regulations of ≤0.3 and ≤0.9 μg/kg, respectively, indicating the suitability of the method for determining trace concentrations of these compounds [17]. Figure 1 depicts the HPLC-FLD chromatograms of the PAH4 standards, PAH4 in the matrix-spiked sample, IS for the blank sample, and the PAHs in a deep-fat fried pork sample.

### 3.2. Comparison of PAHs Content in Deep-Fat Fried Pork Products according to the Frying Conditions

The validated HPLC-FIC method was applied to determine the influence of the frying conditions (type of edible oil, temperature, and time) on the PAH4 levels in deep-fat fried pork. Table 3 presents the levels of the four individual PAHs and PAH4 measured in the pork deep-fat fried in edible oil (soybean, canola, grape seed, and sunflower oil). For this study, deep-fat frying was performed at 160, 180, and 200 °C for 3, 6, and 9 min. The content of BaP in raw pork meat was 0.38 ± 0.26 μg/kg, and the other PAHs were not detected. Deep-fat frying increased the levels of BaP and PAH4. The mean contents of PAH4 in the pork samples ranged from 0.71 to 8.27 μg/kg when deep-fat fried in soybean oil, from 0.90 to 6.33 μg/kg in canola oil, from 0.99 to 9.92 μg/kg in grape seed oil, and from 1.01 to 9.12 μg/kg in sunflower oil, respectively. The highest content of BaP (9.12 ± 0.92 μg/kg) was found in pork deep-fat fried in sunflower oil, followed by grape seed oil (8.35 ± 0.34 μg/kg), soybean oil (8.27 ± 1.35 μg/kg), and canola oil (6.33 ± 0.55 μg/kg), all cooked at 200 °C for 9 min.

The maximum level of BaP was lowest and highest when canola oil (6.33 ± 0.55 μg/kg) and sunflower oil were used (9.12 ± 0.92 μg/kg), respectively. Soybean oil (8.27 ± 1.35 μg/kg) and grape seed oil (8.35 ± 0.34 μg/kg) generated intermediate amounts. This difference in PAH levels emphasizes the influence of the amount of polyunsaturated fatty acid and the differences in the contents of oleic acid, linolenic acid, and linoleic acid in the edible oil in contact with pork during frying.

Three PAHs, including BaA, CHR, and BaP, were detected in all fried pork samples and their concentrations gradually increased as the cooking temperature and time were increased. BbF was not detected in all treatments applied to pork in this study. BaP and PAH4 concentrations significantly increased as cooking time increased for samples fried at the same temperature (*p* < 0.05). Similarly, heat treatment during the cooking process of chicken meat caused a remarkable rise in the total and carcinogenic PAHs content [18]. A study of PAHs in fried fish in Shangdong, China, indicated that the BaP content of fried fish ranged from less than the LOQ to 5.21 μg/kg. Pork has a considerably higher fat content than fried fishery products, and so it can also have a much higher BaP content [19]. Aaslyng et al. concluded that time−temperature was a more important factor than the meat type despite detecting large variations in some of the PAHs in barbecued beef, pork, and chicken, and a markedly higher concentration of PAHs in beef compared to the other meats [20]. Our result provides evidence that some factors (temperature and time) influence the concentrations of PAH4 in deep-fat fried pork products.

### 3.3. Exposure Assessment

The TEQ values of the PAHs are shown in Table 3. The TEQ values of BaP and PAH4 were determined from the PAHs concentration and TEF values (0.10, 0.01, 0.10, and 1.00 for BaA, CHR, BbF, and BaP, respectively) [21]. As a result, the TEQ values of BaP and PAH4 increased proportionally to the time and temperature. The processing condition with the highest TEQ value was deep-fat frying of pork in grape seed oil at 200 °C for 9 min. However, there were similar results between the edible oils examined.

Following estimation of the TEQ value, the exposure assessment of BaP and PAH4 in pork was calculated based on food intake, BW, and exposure duration. Human exposure to BaP and PAH4 was evaluated according to the edible oil for each age group. Age groups were classified as 1–2, 3–5, 6–11, 12–18, 19–29, 30–49, 50–64, and ≥65 years. The results of human exposure by age group are shown in Table 4. Concentrations were analyzed three times, and the average was used. For people of all age groups in Korea, consuming pork products led to average dietary exposures to BaP and PAH4 of 1.67 × 10^−^^4^ and 1.72 × 10^−^^4^ μg-TEQ_BaP_/kg/day, respectively. Additionally, with a high consumption of pork (95th percentile), the average daily exposure to BaP and PAH4 was 8.15 × 10^−^^4^ and 8.54 × 10^−^^4^ μg-TEQ_BaP_/kg/day, respectively.

Although meat intake was high in the age group 19–29 years (45 g/day), those aged 30–49 years presented the highest human exposure to BaP and PAH4. According to Kim et al., ages 3–5 years were the most exposed to BaP in marine products. All factors, such as average daily intake, exposure period, and average weight, contribute to cancer risk, so there is less cancer risk in adults than infants from food [22].

### 3.4. Risk Characterization

Based on the data calculated through the exposure assessment, the MOE to PAH4 for fried pork was obtained using the BMDL and the dietary exposure. The MOE to PAH4 for the consumption of deep-fat fried pork with respect to frying time (3, 6, 9 min) and temperature (160, 180, 200 °C) are shown in Table 5 and Table 6, respectively.

From the dietary exposure results on the analyzed deep-fried pork products, the risk was characterized by calculating the MOE values for Koreans. In the deep-fried pork products, the MOE value for the total population and 95th percentile population ranged from 7.95 × 10^5^ to 3.83 × 10^6^ and from 1.60 × 10^5^ to 7.72 × 10^5^, respectively.

As the frying time and temperature increased, the CDI value increased, and the MOE value decreased. Nonetheless, all of the MOE values indicated a “low concern” (from 10,000 to 1,000,000) or “negligible concern” (>1,000,000) for the total population when compared with those reported by the Committee on Carcinogenicity of Chemicals in Food, Consumer Products and the Environment (COC), which evaluates chemicals for their human carcinogenic potential at the request of UK Government Departments and Agencies [23].

According to Duedahl-Olesen et al., the MOE is 8450 for PAH4 originating from the consumption of home-grilled meat in Denmark [24]. By contrast, the MOE of eight PAHs originating from fishery products in Korea was >1,000,000, demonstrating that intake associated with fishery products was negligible [25]. Comparing these with our results, food with a higher fat content showed lower MOE than food with a lower fat content. In addition, the MOE values by human age according to the frying time and temperature decreased as the age increased up to 50 years. However, the MOE values seem to increase as eating habits change, as digestive ability decreases from the age of 50.

## 4. Conclusions

Previous studies have shown that PAHs can be produced in a variety of processed foods. Dietary exposure and the associated health hazard of PAHs have been investigated in vivo and in vitro. Recent research suggests that PAH4 (BaA, CHR, BbF, and BaP) are the most suitable indicators of carcinogenic risk from PAHs. In this study, we investigated the PAH4 concentration in deep-fat fried pork by cooking condition (temperature and time) and edible oil (soybean oil, canola oil, grape seed oil, and sunflower oil) and evaluated the exposure assessment and risk characterization. The analytical technique of the HPLC-FID method was validated for the investigation of the PAH4 in deep-fat fried pork products. The result of pork products without processing, BaA, CHR, and BbF was <LOQ, and BaP was below 2 μg/kg.

This study provided important information to understand the effect of cooking temperature and time on PAHs formation. In the case of BbF, it was not detected under all frying conditions, and BaA and CHR were increased simply by numerical value but were not significant results. In the case of BaP, it increased significantly as the temperature increased at 3, 6, and 9 min (*p* < 0.05). In addition, when examining the change in BaP over time, the content of BaP significantly increased as the heating time increased in 160, 180, and 200 °C (*p* < 0.05). Thus, total PAHs level in deep-fat fried pork products significantly increased as cooking temperature and time increased (*p* ≤ 0.0003). In addition, in the case of soybean oil, pork products fried at 160 °C for 9 min, 180 °C for 6 min and 9 min, and 200 °C for 3, 6, and 9 min exceeded the “safe criterion” value (2 μg/kg) of BaP set by the European Union. The CDI values for BaP increased as the frying process temperature and time increased due to increased contamination. As a result, the MOE value decreased, indicating a “low risk effect”. The concentration of PAH4 did not exceed the “safe criterion” value set by the European Union, and the MOE values were all over 10,000 in this study. The result can be used as basic data for the PAH4 contents of frying pork and utilized to set legal regulations on PAH4 in Korea.

## Figures and Tables

**Table 1:** HPLC-FLD analysis conditions for the polycyclic aromatic hydrocarbons.

Instrument	Dionex U3000 HPLC		
Column	ZORBAX Eclipse C18 Plus (4.6 mm × 250 mm, 5 μm)		
**Wavelength**		Excitation (nm)	Emission (nm)
	0–20 min	245	390
	20–60 min	294	404
**Mobile phase**		Acetonitrile	Water
	0–20 min	65	35
	20–60 min	70	30
**Flow rate**	1.0 mL/min		
**Temperature**	37 °C		
**Injection volume**	10.0 μL		

**Table 2:** The limit of detection (LOD), limit of quantification (LOQ), linearity, and recovery of each polycyclic aromatic hydrocarbons (PAHs).

PAHs	LOD (μg/kg) ^1^	LOQ (μg/kg) ^2^	Linearity (R^2^) ^3^	Recovery (%) ^4^
Benz[a]anthrancene	0.14	0.42	y = 0.0251x + 0.0043 R^2^ = 0.9989	99.9 ± 1.1
Chrysene	0.10	0.32	y = 0.0176x + 0.0217 R^2^ = 0.9976	95.5 ± 1.4
Benzo[*b*]fluoranthene	0.16	0.50	y = 0.0192x + 0.0060 R^2^ = 0.9975	88.0 ± 0.2
Benzo[*a*]pyrene	0.18	0.55	Y = 0.1340x − 0.0318 R^2^ = 0.9932	91.8 ± 1.4

^1^ Limit of detection based on signal-to-noise ratio as S/N = 3, LOD (μg/kg). ^2^ Limit of quantification based on signal-to-noise ratio as S/N = 10, LOQ (μg/kg). ^3^ Value of the correlation coefficient, R^2^, for the plots recorded in the range of the 10 ng detection limit for each standard. ^4^ Recovery values were determined using 5.0 μg/kg of four different PAHs and shown as recovery ± relative standard deviation.

**Table 3:** Changes in the concentration of PAHs in deep-fat fried pork according to treatment conditions.

Oil	Cooking Temperature (°C)	Time	PAHs (μg/kg) ^1^					TEQ_BaP_ (μg/kg/day)	
		(min)	BaA	CHR	BbF	BaP	PAH4	BaP	PAH4
		0	<LOQ ^2^	<LOQ	<LOQ	0.38 ± 0.26	0.38 ± 0.26	0.38	0.38
Soybean	160	3	<LOQ	<LOQ	<LOQ	0.86 ± 0.14	0.86 ± 0.14	0.86	0.86
		6	<LOQ	<LOQ	<LOQ	1.59 ± 0.52	1.59 ± 0.52	1.59	1.59
		9	0.55 ± 0.07	<LOQ	<LOQ	2.66 ± 0.31	3.21 ± 0.37	2.66	2.71
	180	3	<LOQ	<LOQ	<LOQ	1.50 ± 0.15	1.50 ± 0.15	1.50	1.50
		6	0.14 ± 0.24	<LOQ	<LOQ	2.42 ± 0.14	2.56 ± 0.33	2.42	2.44
		9	0.71 ± 0.07	<LOQ	<LOQ	3.50 ± 0.56	4.21 ± 0.50	3.50	3.57
	200	3	<LOQ	<LOQ	<LOQ	2.66 ± 0.32	2.66 ± 0.32	2.66	2.66
		6	0.17 ± 0.29	<LOQ	<LOQ	3.59 ± 0.12	3.76 ± 0.33	3.59	3.61
		9	0.86 ± 0.09	<LOQ	<LOQ	5.95 ± 2.01	6.81 ± 2.00	5.95	6.03
Canola	160	3	<LOQ	<LOQ	<LOQ	1.03 ± 0.16	1.03 ± 0.16	1.03	1.03
		6	<LOQ	<LOQ	<LOQ	1.63 ± 0.12	1.63 ± 0.12	1.63	1.63
		9	<LOQ	0.14 ± 0.25	<LOQ	3.72 ± 0.58	3.86 ± 0.67	3.72	3.72
	180	3	<LOQ	<LOQ	<LOQ	1.43 ± 0.09	1.43 ± 0.09	1.43	1.43
		6	<LOQ	<LOQ	<LOQ	1.99 ± 0.36	1.99 ± 0.36	1.99	1.99
		9	0.19 ± 0.32	0.18 ± 0.31	<LOQ	2.54 ± 0.42	2.90 ± 0.14	2.54	2.56
	200	3	<LOQ	<LOQ	<LOQ	1.71 ± 0.16	1.71 ± 0.16	1.71	1.71
		6	<LOQ	0.15 ± 0.27	<LOQ	2.95 ± 0.32	3.10 ± 0.59	2.95	2.95
		9	0.23 ± 0.39	0.22 ± 0.38	<LOQ	5.06 ± 1.22	5.50 ± 0.88	5.06	5.08
Grape seed	160	3	<LOQ	<LOQ	<LOQ	1.06 ± 0.09	1.06 ± 0.09	1.06	1.06
		6	<LOQ	0.20 ± 0.34	<LOQ	1.49 ± 0.14	1.69 ± 0.48	1.49	1.49
		9	<LOQ	0.22 ± 0.39	<LOQ	2.52 ± 0.03	2.74 ± 0.40	2.52	2.52
	180	3	<LOQ	<LOQ	<LOQ	1.40 ± 0.17	1.40 ± 0.17	1.40	1.40
		6	<LOQ	0.2 ± 0.35	<LOQ	2.01 ± 0.07	2.21 ± 0.36	2.01	2.01
		9	<LOQ	0.24 ± 0.42	<LOQ	3.80 ± 1.61	4.04 ± 2.03	3.80	3.80
	200	3	<LOQ	0.16 ± 0.28	<LOQ	1.91 ± 0.48	2.07 ± 0.55	1.91	1.91
		6	<LOQ	0.23 ± 0.4	<LOQ	2.95 ± 0.32	3.18 ± 0.67	2.95	2.95
		9	0.27 ± 0.47	0.80 ± 0.78	<LOQ	6.83 ± 1.33	7.90 ± 2.46	6.83	6.86
Sunflower	160	3	<LOQ	<LOQ	<LOQ	1.09 ± 0.07	1.09 ± 0.07	1.09	1.09
		6	<LOQ	<LOQ	<LOQ	1.33 ± 0.20	1.33 ± 0.20	1.33	1.33
		9	<LOQ	0.19 ± 0.32	<LOQ	2.44 ± 1.21	2.63 ± 1.11	2.44	2.45
	180	3	<LOQ	0.16 ± 0.28	<LOQ	1.31 ± 0.07	1.47 ± 0.34	1.31	1.31
		6	<LOQ	0.33 ± 0.28	<LOQ	2.63 ± 0.05	2.95 ± 0.25	2.63	2.63
		9	<LOQ	0.43 ± 0.38	<LOQ	4.41 ± 1.56	4.84 ± 1.79	4.41	4.42
	200	3	<LOQ	0.17 ± 0.29	<LOQ	2.21 ± 0.59	2.37 ± 0.66	2.21	2.21
		6	<LOQ	0.45 ± 0.41	<LOQ	3.42 ± 0.45	3.87 ± 0.68	3.42	3.42
		9	<LOQ	0.37 ± 0.64	<LOQ	6.81 ± 2.48	7.17 ± 3.02	6.81	6.81

^1^ All samples were replicated three times and are expressed with mean ± standard deviation. ^2^ <LOQ = less than lower limit of quantification.

**Table 4:** Comparison of CDI of fried pork by oil type for each group.

Age	Soybean Oil				Canola Oil				Grape Seed Oil				Sunflower Oil			
	BaP (μg/kg)		PAH4 (μg/kg)		BaP (μg/kg)		PAH4 (μg/kg)		BaP (μg/kg)		PAH4 (μg/kg)		BaP (μg/kg)		PAH4 (μg/kg)	
	Mean	95th	Mean	95th	Mean	95th	Mean	95th	Mean	95th	Mean	95th	Mean	95th	Mean	95th
>65	0.32	2.03	0.33	2.06	0.28	1.72	0.28	1.73	0.37	2.33	0.37	2.34	0.37	2.32	0.37	2.32
50–64	0.38	1.90	0.39	1.93	0.33	1.62	0.33	1.62	0.44	2.18	0.44	2.19	0.44	2.18	0.44	2.18
30–49	0.77	3.66	0.78	3.72	0.66	3.11	0.66	3.13	0.89	4.20	0.89	4.23	0.89	4.19	0.89	4.19
19–29	0.53	2.64	0.54	2.67	0.45	2.24	0.45	2.25	0.61	3.03	0.61	3.04	0.60	3.02	0.61	3.02
12–18	0.36	1.61	0.37	1.63	0.31	1.37	0.31	1.37	0.42	1.85	0.42	1.86	0.42	1.84	0.42	1.84
6–11	0.32	1.36	0.33	1.38	0.28	1.16	0.28	1.17	0.37	1.57	0.38	1.57	0.37	1.56	0.37	1.56
3–5	0.18	0.77	0.18	0.78	0.15	0.65	0.16	0.66	0.21	0.88	0.21	0.88	0.21	0.88	0.21	0.88
1–2	0.09	0.52	0.09	0.53	0.08	0.44	0.08	0.44	0.11	0.60	0.11	0.60	0.11	0.60	0.11	0.60

**Table 5:** Variation of MOE value by frying time for each age group.

Age	3 min				6 min				9 min			
	Average Dietary Exposure (μg-TEQ_BaP_/kg/day)		95th Percentile Dietary Exposure (μg-TEQ_BaP_/kg/day)		Average Dietary Exposure (μg-TEQ_BaP_/kg/day)		95th Percentile Dietary Exposure (μg-TEQ_BaP_/kg/day)		Average Dietary Exposure (μg-TEQ_BaP_/kg/day)		95th Percentile Dietary Exposure (μg-TEQ_BaP_/kg/day)	
	BaP	PAH4	BaP	PAH4	BaP	PAH4	BaP	PAH4	BaP	PAH4	BaP	PAH4
>65	9.36 ×10^5^	4.55 ×10^6^	1.50 ×10^5^	7.29 ×10^5^	6.10 ×10^5^	2.96 ×10^6^	9.77 ×10^4^	4.74 ×10^5^	3.50 ×10^5^	1.69 ×10^6^	5.61 ×10^4^	2.71 ×10^5^
50–64	7.90 ×10^5^	3.77 ×10^6^	1.60 ×10^5^	7.63 ×10^5^	5.15 ×10^5^	2.38 ×10^6^	1.04 ×10^5^	4.81 ×10^5^	2.96 ×10^5^	1.29 ×10^6^	5.99 ×10^4^	2.61 ×10^5^
30–49	3.93 ×10^5^	1.91 ×10^6^	8.30 ×10^4^	3.96 ×10^5^	2.56 ×10^5^	1.24 ×10^6^	5.41 ×10^4^	2.50 ×10^5^	1.47 ×10^5^	7.10 ×10^5^	3.11 ×10^4^	1.36 ×10^5^
19–29	5.76 ×10^5^	2.75 ×10^6^	1.15 ×10^6^	5.50 ×10^5^	3.75 ×10^5^	1.73 ×10^6^	7.51 ×10^4^	3.47 ×10^5^	2.15 ×10^5^	9.40 ×10^5^	4.32 ×10^4^	1.88 ×10^5^
12–18	8.38 ×10^5^	4.07 ×10^6^	1.89 ×10^5^	9.19 ×10^5^	5.46 ×10^5^	2.65 ×10^6^	1.23 ×10^5^	5.98 ×10^5^	3.14 ×10^5^	1.51 ×10^6^	7.08 ×10^4^	3.42 ×10^5^
6–11	9.36 ×10^5^	4.54 ×10^6^	2.22 ×10^5^	1.08 ×10^5^	6.10 ×10^5^	2.96 ×10^6^	1.45 ×10^5^	7.04 ×10^5^	3.50 ×10^5^	1.69 ×10^6^	8.34 ×10^4^	4.02 ×10^5^
3–5	1.67 ×10^6^	8.11 ×10^6^	3.97 ×10^5^	1.93 ×10^6^	1.09 ×10^7^	5.28 ×10^6^	2.58 ×10^5^	1.25 ×10^6^	6.25 ×10^5^	3.01 ×10^6^	1.48 ×10^5^	7.16 ×10^5^
1–2	3.28 ×10^6^	1.59 ×10^7^	5.84 ×10^5^	2.84 ×10^6^	2.14 ×10^7^	1.04 ×10^7^	3.80 ×10^5^	1.84 ×10^6^	1.23 ×10^6^	5.92 ×10^6^	2.19 ×10^5^	1.05 ×10^6^

**Table 6:** Variation of MOE value by frying temperature for each age group.

Age	160 °C				180 °C				200 °C			
	Average Dietary Exposure (μg-TEQ_BaP_/kg/day)		95th Percentile Dietary Exposure (μg-TEQ_BaP_/kg/day)		Average Dietary Exposure (μg-TEQ_BaP_/kg/day)		95th Percentile Dietary Exposure (μg-TEQ_BaP_/kg/day)		Average Dietary Exposure (μg-TEQ_BaP_/kg/day)		95th Percentile Dietary Exposure (μg-TEQ_BaP_/kg/day)	
	BaP	PAH4	BaP	PAH4	BaP	PAH4	BaP	PAH4	BaP	PAH4	BaP	PAH4
>65	8.66 ×10^5^	4.20 ×10^6^	1.39 ×10^5^	6.73 ×10^5^	6.20 ×10^5^	3.00 ×10^6^	3.00 ×10^4^	9.94 ×10^5^	4.10 ×10^5^	1.99 ×10^6^	6.58 ×10^4^	3.19 ×10^5^
50–64	7.31 ×10^5^	3.46 ×10^6^	1.48 ×10^5^	7.00 ×10^5^	5.23 ×10^5^	2.40 ×10^6^	1.06 ×10^5^	4.86 ×10^5^	3.46 ×10^5^	1.58 ×10^6^	7.01 ×10^4^	3.21 ×10^5^
30–49	3.64 ×10^5^	1.76 ×10^6^	7.68 ×10^4^	3.63 ×10^5^	2.60 ×10^5^	1.26 ×10^6^	5.50 ×10^4^	2.52 ×10^5^	1.72 ×10^5^	8.36 ×10^5^	3.64 ×10^4^	1.66 ×10^5^
19–29	5.32 ×10^5^	2.52 ×10^6^	1.07 ×10^5^	5.05 ×10^5^	3.81 ×10^5^	1.75 ×10^6^	7.64 ×10^4^	3.50 ×10^5^	2.52 ×10^5^	1.15 ×10^6^	5.06 ×10^4^	2.31 ×10^5^
12–18	7.75 ×10^5^	3.76 ×10^6^	1.75 ×10^5^	8.49 ×10^5^	5.55 ×10^5^	2.69 ×10^6^	1.25 ×10^5^	6.07 ×10^5^	3.67 ×10^5^	1.78 ×10^6^	8.29 ×10^4^	4.02 ×10^5^
6–11	8.66 ×10^5^	4.20 ×10^6^	2.06 ×10^5^	1.00 ×10^6^	6.20 ×10^5^	3.00 ×10^6^	1.48 ×10^5^	7.15 ×10^5^	4.10 ×10^5^	1.99 ×10^6^	9.78 ×10^4^	4.74 ×10^5^
3–5	1.54 ×10^6^	7.49 ×10^6^	3.67 ×10^5^	1.78 ×10^6^	1.11 ×10^6^	5.36 ×10^6^	2.63 ×10^5^	1.27 ×10^6^	7.32 ×10^5^	3.55 ×10^6^	1.74 ×10^5^	8.43 ×10^5^
1–2	3.03 ×10^6^	1.47 ×10^7^	5.40 ×10^5^	2.62 ×10^6^	2.17 ×10^6^	1.04 ×10^7^	3.87 ×10^5^	1.88 ×10^6^	1.44 ×10^6^	6.97 ×10^6^	2.56 ×10^5^	1.24 ×10^6^

## Data Availability

Not applicable.
